# Characteristics, Usability, and Users Experience of a System Combining Cognitive and Physical Therapy in a Virtual Environment: Positive Bike

**DOI:** 10.3390/s18072343

**Published:** 2018-07-19

**Authors:** Elisa Pedroli, Luca Greci, Desirèe Colombo, Silvia Serino, Pietro Cipresso, Sara Arlati, Marta Mondellini, Lorenzo Boilini, Valentina Giussani, Karine Goulene, Monica Agostoni, Marco Sacco, Marco Stramba-Badiale, Giuseppe Riva, Andrea Gaggioli

**Affiliations:** 1 Applied Technology for Neuro-Psychology Lab, I.R.C.C.S. Istituto Auxologico Italiano, 20149 Milano, Italy; silvia.serino@unicatt.it (S.S.); pietro.Cipresso@unicatt.it (P.C.); giuseppe.riva@unicatt.it (G.R.); andrea.gaggioli@unicatt.it (A.G.); 2 Industrial Technologies and Automation, Consiglio Nazionale delle Ricerche, 20133 Milano, Italy; luca.greci@itia.cnr.it (L.G.); sara.arlati@itia.cnr.it (S.A.); marta.mondellini@itia.cnr.it (M.M.); marco.sacco@itia.cnr.it (M.S.); 3 Department of Basic Psychology, Clinic and Psychobiology, Universitat Jaume I, Av. Sos Baynat, s/n, 12071 Castellón, Spain; colombo.dsr@gmail.com or dcolombo@uji.es; 4 Department of Psychology, Università Cattolica del Sacro Cuore, 20123 Milano, Italy; 5 Department of Electronics, Information and Bioengineering, Politecnico di Milano, 20133 Milano, Italy; 6 Department of Geriatrics and Cardiovascular Medicine, I.R.C.C.S. Istituto Auxologico Italiano, 20149 Milano, Italy; lo.boilini@gmail.com (L.B.); v.giussani@auxologico.it (V.G.); goulene@auxologico.it (K.G.); stramba_badiale@auxologico.it (M.S.-B.); 7 Nursing Home Monsignor Bicchierai, I.R.C.C.S. Istituto Auxologico Italiano, 20149 Milano, Italy; m.agostoni@auxologico.it

**Keywords:** virtual reality, rehabilitation, ageing, frailty, usability, UX

## Abstract

We present the architecture and usability evaluation of virtual reality system—“Positive Bike”—designed for improving cognitive and motor conditions in frail elderly patients. The system consists of a cycle-ergometer integrated in an immersive virtual reality system (CAVE) which allows combining motor and cognitive exercises according to a “dual-task” paradigm. We tested the usability and user’s experience of the prototype in a pilot evaluation study that involved five elderly patients. The prototype was tested in one-session training to understand the limitations and areas for improvement of our system. The evaluation consisted in (i) usability assessment using the system usability scale; (ii) evaluation of user’s engagement using the flow state scale; and (iii) expert evaluation involving interviews with domain experts. Results showed a good usability, both for system usability scale and the semi-structured interview. The level of flow (i.e., enjoyment with the task at hand) measured using the short flow state scale, was also high. Analysis of semi-structured interview carried out with domain experts provided further indications to improve the system. Overall, these findings show that, despite some limitations, the system is usable and provides an enjoyable user’s experience.

## 1. Introduction

### 1.1. The Problem of Frailty

Aging is a physiological process involving both cognitive and motor domains, and affecting many aspects of everyday life. According to the World Health Organization, the proportion of people older than 60-year-old is increasing rapidly and faster than all the other age groups [1]. In the population, in the last decade, there has been a lot of interest in “frail” patients, constituting the 6.9% of adults older than 65-year-old [2]. Specifically, frailty is a clinical condition and a state of vulnerability associated with increasing age and affecting multiple domains such as gait, mobility, balance and cognition [3]. According to the standardized definition of Fried and colleagues, three or more of the following criteria should be met: unintentional weight loss (10 lbs in past year), self-reported exhaustion, weakness (grip strength), slow walking speed, and low physical activity [2]. Evidence of a strong association between this condition and higher risks for adverse health outcomes, such as mortality, disability and, especially, high risk of falls, has been shown [2,4,5,6].

Even though cognitive and motor impairments have been considered and treated independently, literature is showing evidence for a strong relation between them, both in healthy and pathological conditions. An example of this relationship is the risk of falls. Among old adults and frail patients, falls are one of the most critical public health problems, as well as the major cause of injuries: one in three old people, indeed, falls at least once in a year [7], with subsequent consequences in terms of loss of independence and adverse psychosocial problems [8,9]. The increased fall rate among older adults has been interpreted in light of the cognitive-motor interference (CMI) theory [10,11].

CMI, a specific type of dual task interference (DTi), refers to the simultaneous execution of a cognitive and a motor task, that requires a great amount of cognitive control in terms of executive functions and attentional abilities [12]. The concurrent performance of a cognitive task can cause a decline either in the motor or in the cognitive execution, or even in both, depending on the cognitive demand [1,12,13]. Current literature has shown that the mechanisms supporting DT are still unclear. As a matter of fact, a specific brain structure devoted to the control of DT has not been yet identified: rather than being a simple addictive effect, DT could be the result of a complex coordination and interplay between different specialized information-processing systems [14]. Concerning the cognitive mechanisms, instead, two different attentional theories have been proposed. Along with the Wickens’ theory of shared attentional resources [15], the concurrent execution of two activities would require to divide and re-allocate attention, thus decreasing the attentional resources assigned to each single task [16]. On the other hand, the bottleneck hypothesis argues that the main cause of interference would be the competition for information-processing in neural pathways [16]: tasks that are supported by a similar neural network could not be carried out in parallel, but only in sequence.

Interestingly, successful locomotion requires the ability of performing simultaneously a cognitive task that can cause an interference in gait performance, especially in older adults. Several works showed the efficacy of this paradigm [17,18,19,20]. The age-related decrease in attentional and executive functioning would impair the ability of managing the concurrent execution of different motor and cognitive activities, normally occurring in everyday life [21,22]. Notably, frailty has been described as a reversible dynamic process, characterized by recurrent transitions between states over time [23]. As a consequence, a growing number of studies focuses on the possibility of creating specific interventions, either to improve or prevent frailty and, specifically, to reduce the risk of fall [24]. For instance, regular physical exercise and motor interventions, either in their aerobic or strength form [25], were proved to bring many benefits for reducing the fall risk [26,27,28,29] and improving general cognitive functioning [30]. Accordingly, a recent systematic review showed the main role played by muscular strength and postural balance for the prevention of falls [28].

Recently, DT has been suggested as a more efficient approach for the improvement of cognitive and motor performances [21,31,32,33]. Specifically, the important contribution of high-order cognitive systems in gait control would make DT an effective training for the reduction of fall risk [21].

### 1.2. The Potential of Virtual Reality to Counteract Frailty

Thanks to the development of new technologies and to the great diffusion of virtual reality (VR) in the clinical field, it is now possible to develop and implement interactive cognitive-motor training. VR offers indeed the opportunity to create ecological and realistic environments in which to reproduce daily-life situations, leading to higher acceptance and adherence rates among patients [34]. In addition, VR allows rehabilitation practice to become more engaging, thus increasing users’ motivation and performances; finally, VR—coupled with appropriate sensors—enables measuring different objective behaviors in real-time, thus allowing the provision of training in a valid, safe, and controlled environment. 

The adoption of VR training, mainly involving balance and functional mobility, has already shown promising outcomes in the clinical field, thus suggesting VR as an appropriate complementary approach in the field of rehabilitation [35]. With respect to elderly rehabilitation programs, and the implementation of fall prevention exercise, many VR-based studies can be traced in literature [36]; among them, the use of balance boards and the implementation of a balance training program using commercial games from the Wii Fit software package (i.e., yoga, soccer, ski jump, tennis) are quite common [37,38,39]. In the majority of the cases, VR-based balance programs resulted in improved postural balance and fear of falling in the experimental groups when compared to no treatment [36]. Other setups foresee the exploitation of step pads and/or of the Kinect sensor, and the provision of visual feedback on a TV screen to support the maintenance of the adequate step rhythm [40] or the awareness of quality of the performance [41]. A recent study investigated the effects of the addition of a non-immersive virtual reality component to treadmill training aimed at reducing fall risk in older adults [42]; in this case, VR was used to increase the ecological validity of the training, providing older adults with real-life challenges such as obstacles and distractors requiring continual adjustment of steps. The whole system included the treadmill, a safety harness, a virtual environment displayed on a TV screen, and a Kinect sensor tracking the participants’ steps. Six months after the end of the program, the group who underwent the training using treadmill reported a significant decrease in fall incident rate, with respect to the group who trained with treadmill alone. 

Several studies already adopted a VR cycling training for the motor rehabilitation of old adults or stroke patients [43,44,45,46,47], but no one ever implemented it into a DT protocol, thus requiring the execution of some interactive cognitive tasks during the physical performance of the virtual ride. To our knowledge, studies exploiting VR-based dual task training focused on exercises requiring locomotion and involved either the use of a treadmill [48,49] or implemented stepping-in-place on a balance board [19]. In the following, we describe the rationale, the design and the usability testing of ‘Positive Bike’, a fully-immersive VR biking experience for the implementation of an interactive DT training. Stationary cycle exercises can improve balance, weight shifts and gait, as well as lower body extremity functioning, thus translating into a significant reduction of the fall risk [46,50,51]. The pattern of cycling is indeed very close to walking, as they are both cyclical, they both involve the reciprocal flexing and extension movements from the hip, knee, and ankle, and they both activate alternatively agonist and antagonist muscles [52,53,54]. Moreover, the use of a stationary bike results in providing the user with a controllable workload and a safer equipment; indeed, with respect to the treadmill (the other equipment allowing an easy modification of the workload), the employment of a cycle-ergometer is associated with a lower risk of injury, especially in case of elderly and frail users [55]. 

Another key requirement was to create a task that provided participants with positive and engaging experience. According to Riva and colleagues [56,57] a key asset of VR for rehabilitation is that this technology allows creating artificial environments that promote optimal experience through surprising psychological resources and increase in the involvement. Accordingly, VR is a powerful tool that can be used to improve the engagement of the participants, thanks to the creation of challenging tasks designed accordingly to the user’s personal skills and resources. This approach, also called “transformation of flow” has shown promising results in the field of rehabilitation, both cognitive and physical [58,59].

## 2. Positive Bike

### 2.1. System Architecture

The system is constituted by a cycle-ergometer (Cosmed Eurobike 320), a pushing button anchored on the cycle-ergometer handlebars, an Arduino2 board connecting the button to the computer and an Xbox controller. All these components are placed inside a Cave Automatic Virtual Environment (CAVE), a room-sized cube in which the 3D visualization of the virtual environments (VEs) occurs thanks to the combination of four stereoscopic projectors (Full HD 3D UXGA DLP), three rear-projection screens (i.e., the three walls) and one downward-projection screen, all having a projectable area of 266 × 200 cm. A cluster system composed of two HPZ620 Graphics Workstations, mounting Nvidia Quadro K6000 GPU with dedicated Quadro Sync cards, is responsible for the rendering of the four projection surfaces, user tracking and functional logic. CAVE is in fact equipped with a Vicon motion tracking system, with four infrared cameras with 1-megapixel resolution, which allows the tracking of specific reflective markers positioned on target objects and a correct reading of the simulated spaces and distances with a 1:1 scale ratio, thus enhancing the feeling of being immersed in the virtual scene. In order to improve the safety of patients, we decided to implement a cycle-ergometer-based training inside the CAVE, instead of using a treadmill.

In the case of this study, passive markers are used to track the position of the shutter glasses the user has to wear during the exercise in order to change the point-of-view in the VE accordingly. In addition, markers are used to track the heading of the X-box controller; this allows operators to use the X-box controller as a laser-pointing device and to select the interactive contents projected on the screens (e.g., buttons, toggles, etc.) pushing the “A” button on the controller. This input device is not fixed within the system. It is brought in when needed, for example to select the exercise parameters. Conversely, it is brought out of the system when it is no longer useful, for example during the exercise with the cycle-ergometer.

The cycling velocity, as well as the workload, can be read and set thanks to an ad hoc communication protocol developed exploiting the cycle-ergometer software development kit (SDK) provided by the manufacturer; the bike is connected to the computer via a serial cable, as well as the Arduino board.

**Figure 1:**
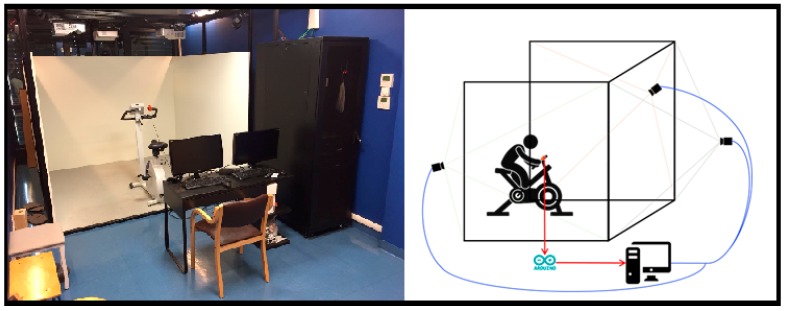
A picture of a real set up and a schematic representation of the hardware setup.

The VE has been designed and implemented using Unity3D and displayed in the CAVE using MiddleVR for Unity (http://www.middlevr.com/middlevr-for-unity/). This Unity plug-in provides driver mappings for a variety of existing input devices and accessories—such as Vicon trackers—and delivers abstractions to split functional and graphical logic into a clustered stereoscopic multi display setup, allowing for multi-screens/multi-computers synchronization for higher-resolution VR systems. The hardware setup is shown in Figure 1.

### 2.2. Virtual Environments

**Figure 2:**
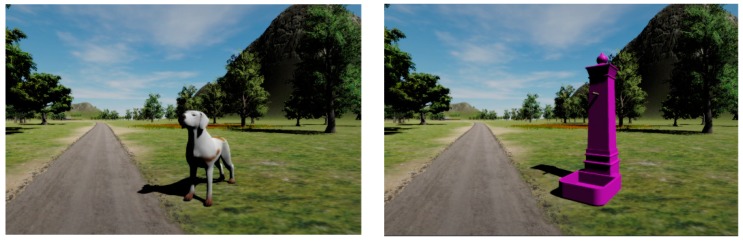
Examples of the two types of targets: a dog and a colored fountain.

The Positive Bike application allows for the accomplishment of dual task training, by providing both cognitive and motor tasks at the same time. The motor exercise will be described later in the article. There is the possibility to choose two different types of cognitive exercise (i.e., using “animals” or “street furniture”) while cycling. In both cases, the cognitive task consists in identifying a series of target objects appearing by the side of the pathway, according to two different criteria. If players are exercising using animals, at the beginning of the exercise, each player is assigned a letter and he/she is asked to identify all the animals whose name starts with that letter (e.g., for the letter “d”, “dog” is a target, whereas “horse” is not). Instead, if the target objects are constituted by street furniture, each player is assigned a color (Figure 2).

More in details, Positive Bike VE is composed of three different scenes which are displayed in the following order: (1) login and settings, (2) exercise, and (3) attention test. The login and settings scenes are populated with a 3D graphical user interface (GUI) allowing the therapist either to create a new user or to load the information of an already existing user. Moreover, it permits the setting of the parameters defining the exercise, which are:

- Game type: the therapist can set the target typology—and thus define the exercise type—by choosing between animals or street furniture.
- Characteristic of the target to select: for animals, the first letter of the animals’ names (C/G/T/S); for street furniture, distinct colors are available (orange/blue /yellow/violet).
- Level: two levels of difficulty are available; in level 1, targets appear on the route each 15 s, in level 2, each 10 s.
- Cycle-ergometer workload: the operator can set the bike workload selecting among 20/30/40/50 Watt, depending on the patient’s physical condition.
- Time: the duration of the exercise, the therapist can select 15 or 20 min.

The selection of the user and of the training parameters are carried out with the X-box controller by the therapist. After the definition of such parameters, he/she can start the exercise.

The exercise scene contains a trail in the park that flows according to the pedals velocity (measured by the cycle-ergometer in revolutions-per-minute, RPM). The path is created thanks to the placement of subsequent nodes on the route, whose interpolation occurs in real-time using quaternion spherical linear interpolation (slerp). Due to the mechanical characteristics of the cycle-ergometer, the user cannot brake or turn intentionally. Being aware of these limitations, the park has been designed to limit any possible desire to deviate from the predefined path. The trail is one and only, and no road forks are present; however, to avoid boredom, elements of the landscape change throughout the exercise, i.e., different species of plants and trees, lakes, buildings, etc. appear on the background. Very slight bends are present to increase the realism of the scenario; prior to the execution of the pilot study, tests on healthy subjects ensured that such curvatures do not induce cyber-sickness due to the expectation of lateral accelerations [60].

To elicit an appropriate level of physical effort, participants are instructed to keep their cycling velocity between 55 and 65 RPM: the exercise intensity is adjusted by the therapist who can choose among different ergometer workload values according to the subjects’ capabilities. Two different audio warnings are used to provide the users with feedback when the velocity is too high or too low. In particular, an acute sound indicates that the user is cycling too fast, whereas a grave one signals a too low velocity. Both feedbacks are given in the form of earcons [61]: this type of auditory display, defined as “abstract and synthetic tones”, were preferred to visual indications because they provide an immediate feedback [62] without requiring the patients to look away from the path, thus avoiding the possible missing of a target on the way. During the whole exercise, a rustle simulating the cycling on an untarmacked road is reproduced [63].

During the cycling, the targets appear randomly, on either the left or right side of the street at a distance of 20 m from the user position, so that the participant has the time to clearly distinguish its appearance and features. Targets’ orientation is random, too. The time elapsing between two subsequent apparitions is driven by the difficulty level selected.

To select a target the user has to push the button positioned on the cycle ergometer handlebar (Figure 1), before the target gets out of his/her visual field (i.e., it is not displayed on the CAVE lateral wall anymore). Visual feedback is given to the user both for wrong (target becomes red) or right answer (target becomes green). No feedback is given when the user does not press the button, either if the choice is correct (the displayed animal/object is a distractor) or if the target has been missed.

At the end of the exercise, in scene (3), the user is administered an attention question: the application asks the player—via written text—how many targets he/she encountered during the exercise. The user tells the therapist how many targets he/she remembers. The result of this query inserted in the system by the operator, together with session data (date, time, and duration), exercise parameters and user’s performance (number selected/missed targets) are stored in the user’s folder in the form of an XML file.

## 3. Usability Study

Usability can be defined as the degree to which a specific subject is able to use a given system to achieve specific goals effectively, efficiently, and satisfactorily within a well-defined context of use [64]. According to this definition, usability is composed by three main factors, all related to the characteristics and the goals of the users and the context of use:

- Effectiveness: the possibility for the users to achieve goals;
- Efficiency: the effort made by the user to reach the goal;
- Satisfaction: what users think about the interaction with the system.

Formative evaluation is a process for the assessment of the usability in order to understand what the usability problems are and suggest developers’ solutions to address work according to expert perspective. In the present study, a formative evaluation was carried out using three validated instruments: The system usability scale (SUS) [65], an Italian adaptation of the short flow state scale [66] and a formative evaluation carried out through a semi-structured interview:

- SUS is a “quick and easy to use” questionnaire composed by ten items and created by Brooke in the 1996 [65]. The final score can range from 0, lack of usability, to 100, best usability (for an interpretation of SUS scores, see [67]). This is a standard scale for the assessment of usability of technological systems and it is easy to use and to understand for the patients.
- The short flow state scale [66] assesses nine key flow dimensions: (1) challenge–skill balance: “I feel I am competent enough to meet the high demands of the situation”; (2) action–awareness merging: “I do things spontaneously and automatically without having to think”; (3) clear goals: “I have a strong sense of what I want to do”; (4) unambiguous feedback: “I have a good idea while I am performing about how well I am doing”; (5) concentration on the task at hand: “I am completely focused on the task at hand”; (6) sense of control: “I have a feeling of total control over what I am doing”; (7) transformation of time: “The way time passes seems to be different from normal”; (8) loss of self-consciousness: “I was not worried about what others may have been thinking of me”; (9) and autotelic experience: “The experience is extremely rewarding”. These characteristics were constructed using the conceptual flow model [68,69]. Subjects have to rate the flow experience on a five-point Likert scale ranging from 1 (strongly disagree) to 5 (strongly agree).

The analysis of the transformation of flow could be helpful in evaluating the degree of engagement of patient during the task and, indirectly, the perceived sense of control.

The aim of the “formative evaluations” is to collect information about the usability and interaction from the point of view of the final users. The interview focused on four primary areas: (1) usability; (2) sense of presence; (3) cyber sickness and; (4) expectations. For the first two of these topics, minor themes were identified. In the first case, three systems’ characteristics that give further information about usability were explored; in the second case, three variables that contribute to form the multidimensional construct of sense of presence have been examined in depth:

1. Usability:
  ○ Utilization (effectiveness),
  ○ Learning (efficiency),
  ○ Pleasantness (satisfaction).
2. Sense of presence:
  ○ Spatial presence,
  ○ Engagement,
  ○ Realism.

In the Table 1 some exemplificative questions are reported.

The outcome is a description of the main difficulties emerged during the user of application, the impact of the problem on the usability and the practical solutions. The results of the analysis could be used to refine the interaction design.

### 3.1. Sample

For the usability assessment, five elderly subjects were recruited—three females and two males. The mean age was 70 (SD 11.70) and the mean years of education (y.o.e.) were 11 (SD 5.61). All the demographic data are reported in the Table 2.

Before the session, all participants were given written information about the study and were asked to give written consent to be included. The study received ethical approval from the Ethical Committee of the Istituto Auxologico Italiano.

Subjects with physical problems that prevent riding or with dementia (MMSE < 20) were excluded from the study, no other exclusion criteria were considered. No other cognitive tests have been used because they were considered not related to the usability assessment.

### 3.2. Task

Each subject had to perform the same exercise inside the CAVE. The task required to ride the cycle-ergometer for 15 consecutive minutes inside the virtual environment. The cycle-ergometer workload was set at the minimum level (20W) for all the subjects. The eyeglasses that commanded the visual feedback were hung on the neck of the patients, who had to wear spectacle goggles; this was done to prevent subjects from experiencing cybersickness, such as nausea or dizziness, as while pedaling their head could swing excessively and cause abrupt movements in the virtual environment. Subjects had to keep constant speed during the task. They received an audio feedback if the speed got out of the parameters (Section 2.1). Everyone used animals beginning with letter “C” as target (camel, dog, kangaroo, horse and deer—respectively cammello, cane, canguro, cavallo, and cervo in Italian) and the lowest difficult level (Level 1).

## 4. Results

**Figure 3:**
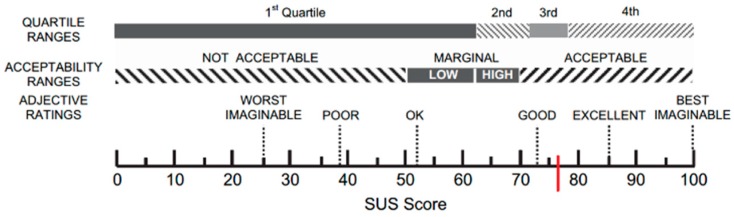
A graphic representation of the SUS’ score.

The mean value of the usability, calculated with the SUS, was 76.88 (SD = 17.00) as showed with a red line in Figure 3.

A global score of flow was obtained calculating the mean of each score of the nine items of the short flow state scale. The mean score for all participants was 4.33 (SD = 0.84); the maximum score of each sub-scale is 5. A single dimension mean score is presented in Table 3.

The outcome of formative evaluation was divided into two tables for clarity. To assess the valence of each feedback, two independent and external judges were involved. They divided the feedbacks in “positive”, or “negative”. Then, the agreement between the judgments was calculated using Cohen’s k coefficient. The results (Table 4) showed an elevated level of agreement between judges (k = 0.85; SE = 0.1). In Table 5, the positive feedbacks of the subjects are reported; the negative ones are presented in Table 6. Direct comments of the subjects are reported in quotation marks, unlike the researchers’ observations.

## 5. Discussion

The results reported were very encouraging and showed that the system had good usability.

The SUS score [57] was 76.88 (SD = 17.00) and indicated a satisfactory level of usability, according to the questionnaire’s score acceptability ranges, grade scale, and adjective ratings [67]: indeed, suc score can be included in the third quartile, as showed in the Figure 3. According to this test, no adaptation of our system would be necessary. 

The innovative design of the system did not allow for the comparison with similar systems, especially in terms of user’s experiences. Previous works, in fact, were mainly focused on the effectiveness of the proposed interventions in reducing falls. In terms of usability evaluation, only one study involving a cycle-ergometer-based system has been traced in literature. Holland et al. [70] developed a system exploiting a tablet and allowing chronic obstructive pulmonary disorders (COPD) patients to participate in a videoconference with both other users and the therapists while cycling. In the study, conducted enrolling eight patients, the obtained median value for usability was 81 [70]. This result belonged to the same acceptability range with respect to the presented system, though it is indeed a little higher. This discrepancy may be explained taking into consideration both the higher simplicity of a tablet-based system with respect to the CAVE environment, and the active participation of the other users in the sessions, which promoted an increased perception of the system’s usability [71]. State of flow induced by the developed system was comparable with the flow generated by Kinect-based exergames aimed at reducing falls in a sample of adults 65+ [72] and higher when compared to a Kinect-based dual task intervention developed for balance improvement in patients with Parkinson’s disease [73] and of an exergame developed with the Wii Fit board dedicated to multiple sclerosis patients [58]. 

Despite the obtained positive results, several issues that could be improved emerged during the formative evaluation. As showed in Table 3, subjects highlighted some features that, in their opinions, could be modified to improve the quality and the usability of the system.

Most patients reported problems in recognizing animals. In some cases, the problem was related to the dimension of the target. The smaller the animal was, the more patients had trouble recognizing it. To solve this problem, a minimum size for all animals could be established, even if this would involve a less realistic choice. Alternatively, other animals, that have not been inserted in this test because they were difficult to recognize or easily confused with others (for example, pricket, goat, stork; in Italian: cerbiatto, capra, cicogna), could be inserted after a special training with patients to make them familiarize with the animals’ appearance. In other cases, the difficulty was related to the way the animals were presented. If the animal was presented backward some subjects had some problems to recognize it correctly. A simple way to fix this problem is to constrain the rotation of all the animals along the path and make them always face the subject.

Two subjects confused some similar animals, i.e., they called the “horse”, a “zebra” and the “swan”, a “turkey”. A training preceding the exercise with the purpose of familiarizing with animals can be of help in this case too. In addition to this, a general improvement of the quality of the 3D animal models could enhance their recognizability and, thus, the usability of the virtual environment.

A problem related to the discrimination of the audio feedback used for the regulation of the riding speed emerged during the sessions. As said before, an acute tone indicated a too-high speed; conversely, a grave tone indicated a too-low speed. These two earcons overlapped the realistic sound of the bike riding that was provided with the aim of improving the realism, the sense of control and the agency in the environment. One subject had difficulty in differentiating the sounds, especially at the beginning of the exercise. Adding a training phase before the exercise, during which subjects could listen to the different sounds and learn to discriminate them, could be an easy-to-use solution to avoid this problem.

In several occasions, a problem with the button showed up: patients pushed it, but the system did not respond. This technical difficulty could be accommodated by adjusting or replacing the button.

The analysis of flow resulted in a very high mean score: 4.33/5 (SD = 0.84). This indicated that subjects were very involved in the environment and in the task. Csikszentmihalyi [69] described flow as a sensation that people feel when they act with total involvement. Flow could be also strongly related to wellbeing, because it emerges when good balance between challenge and personal skills is present, that is a situation characterized by high sense of control. As reported in Table 2, a subject indicated that during the task, she forgot the presence of the examiners. Another subject even perceived that the frequency of appearance of animals had increased, when in reality it was always the same (Table 2). Analyzing the single dimensions of the scale, it is possible to identify the dimension with the major and minor score. The lowest mean score (3.8) but with the highest SD (1.69) was related to the item “The way time passed seemed to be different from normal” that investigate the “transformation of time”. A high SD indicates a greater variability and lower agreement in the answers among subjects. The totality of subjects responded with the maximum rate (5) at the item “I was not worried about what others may have been thinking of me”. This specific sentence is related to the “loss of self-consciousness” dimension. The subjects forgot the context in which they were during this exercise: they forgot to be in a hospital for a rehabilitation program. This aspect could be a strength of the developed system because it may encourage the patients to be part of the rehabilitation sessions and increase their adherence to the program.

## 6. Conclusions and Future Works

VR represents a promising technology that, in the near future, can be easily become part of different rehabilitation treatments, as demonstrated by the great number of studies reported in literature [36,43,58,59]. However, before its introduction in the clinical practice, it is necessary to consider both the pathology-related complications that potential users may have and the usability aspect of the designed system.

A preliminary version of this system was presented in a previous conference paper [74].

In this work, an innovative system for motor rehabilitation was presented together with an ad hoc VE developed for the provision of a dual-task exercise to frail patients. The first usability study—conducted on five elderly subjects—with the aim of assessing the system usability and the end users’ satisfaction resulted in a good level of usability, although limited due to small sample.

A strength of our system was the high level of flow showed by the participants associated with high immersion (Q7—Loss of self-consciousness (mean 5/5; SD = 0.00)) and fun during the experience. This indicates that the system is able to engage the subjects more than a classical training program. This is an important factor to consider when designing interactive systems in the medical and rehabilitative field, as greater involvement leads the patient to achieve better results [75].

However, different issues related both to software and hardware have been highlighted both by patients and by operators observing the training sessions. These issues will be corrected before the next trial and a training phase, during which the patient is instructed about the tasks and the types of feedback, will be arranged. 

Moreover, a limitation of this work is the restricted number of participants involved in the experiment, this problem reduces the generalizability of the results to other subjects. Nevertheless, the homogeneity of the data that emerged from the interviews allows us to hypothesize that many other critical factors with a greater number of subjects would not emerge. 

The clinical trial designed to reduce the risk of falling in frail patients is ongoing. The aim of this study is to compare classical and virtual rehabilitation. For the evaluation of this physical rehabilitation program, only motor outcome measurements are considered. The inclusion criteria of the clinical sample are designed to exclude patients with cognitive impairment (MMSE < 26). The clinical trial includes 10 biweekly sections of bicycle and balance exercises of about 30 min for both classical and virtual rehabilitation. The VR program includes another balance task where patients have to avoid rocks flowing to them in a virtual environment. An important part of such clinical trial includes usability and acceptability assessment, since these variables could help us to improve the generalizability and the strength of these preliminary and partial results.

Furthermore, it would also be interesting to compare the performance and the involvement of patients with this system and with another device, particularly with a head mounted display (HMD). An HMD, in fact, could enhance the navigational experience in the VEs and increase the sense of presence, thought it may have the drawback of inducing cyber-sickness [66]. Future works include also the integration of sensors to monitor the patient physiological status during the training. A heart rate monitor or a breath rate monitor could be easily integrated in the setup to ensure patients’ safety throughout the training sessions and to measure their potential progress during the program.

## Figures and Tables

**Table 1:** Questions of semi-structured interview.

Topic	Sub-Topic	Questions
Usability	Utilization	What difficulties did you encounter in carrying out the task? Was it difficult to use the instrument? There were technical issues during the session?
	Learning	Did you have to ask for help to understand how to use the system? Did it take a long time to figure out how the instrument works? Was exercise complicated?
	Pleasantness	Did you like the virtual environment? Some parts of the system were uncomfortable? Did you have any trouble riding a stationary bike with 3D glasses?
Sense of Presence	Spatial presence	Did you feel part of the environment? Do you feel you have control over the environment?
	Engagement	Were you happy that the exercise was over? What do you think about the duration of the experience? Did you easily get distracted during exercise?
	Realism	How did you find the environment, realistic or too artificial?
Cyber Sickness	Physical side-effects	Did you feel bad during exercise? Did you have nausea, dizziness or other physical symptoms during exercise?
Expectations		Would you like to use this system to do exercise? Do you think this system can be useful for other types of patients?

**Table 2:** Demographic data.

	Age	y.o.e.	Gender	MMSE
Subject 1	87	5	M	27
Subject 2	65	16	F	25.2
Subject 3	77	5	M	20.9
Subject 4	59	16	F	25.2
Subject 5	62	13	F	30
Mean	70.00	11.70		25.66
SD	11.70	5.61		3.31

**Table 3:** Short flow state scale single dimension mean score.

Dimension		Mean	SD
Q1	Challenge-skill	4.6	0.49
Q2	Action-awareness	4	1.26
Q3	Clear goals	4.4	0.80
Q4	Unambiguous feedback	4.2	0.98
Q5	Concentration	4.4	0.80
Q6	Sense of control	4.4	0.80
Q7	Loss of self-consciousness	5	0.00
Q8	Transformation of time	3.8	1.60
Q9	Autotelic experience	4.2	1.60
Q10	Total	4.33	0.75

**Table 4:** Results of agreement analysis.

		Value	Std. Err.
Measure of agreement	Kappa	0.850	0.102
No. of valid cases		27	

**Table 5:** Positive feedbacks from formative evaluation.

Topic	Sub-Topic	Positive Feedback
Usability	Utilization	“Both the motor and cognitive tasks were easy.”
	Learning	“There was no problem in learning the use of the system.”
	Pleasantness	“The 3D glass was not uncomfortable.” “The environment was beautiful.” “The cycle-ergometer was manageable.”
Sense of Presence	Spatial Presence	“The feeling was to be in the real park.” “I had the feeling of being suspended.” “The environment was relaxing.”
	Engagement	“I was focused on the task.” “I think I’ve been pedaling for 5 min.” “I forget you (the examiners) were here too.”
	Realism	“The environment was realistic.”
Cyber Sickness	Physical side-effects	None present side effect like cyber-sickness or nausea
Expectations		“This system could be useful for several types of patients.” “I think it’s easier to train with this tool.”

**Table 6:** Negative feedback from formative evaluation.

Topic	Sub-Topic	Negative Feedback
Usability	Utilization	“It’s difficult to recognize small animals.” “It’s not easy to identify animals placed backward.” “Some similar animals were confused (zebra–horse and turkey–swan).”
	Learning	The sound of the bike might be confused with the sound that give a feedback about speed. “When frequency increases the exercise becomes more difficult.”
	Pleasantness	“Animals are repetitive.”
Sense of Presence	Spatial Presence	“I had the feeling that animals bumped me.”
	Engagement	“I felt passive and not active in the environment.”
	Realism	“The environment was nice but did not look very real.” “Some animals are ‘out of context’.”
Cyber Sickness	Physical side-effects	One patient was tired before the end of the task.
Expectations		There is no difference between this type of treatment and another.
